# YAP-mediated glycolysis promotes pulmonary arterial smooth muscle cell proliferation in pulmonary arterial hypertension

**DOI:** 10.1016/j.jbc.2025.110836

**Published:** 2025-10-16

**Authors:** Wenhua Shi, Liping Chen, Wei Zhang, Ping He, Yonghong Zhang, Kecheng Yan, Cong Li, Pu Ning, Yuan Liu, Shuzhen Ma, Rui Ke

**Affiliations:** 1 Department of Respiratory and Critical Care Medicine, The Second Affiliated Hospital of Xi’an Jiaotong University, Xi’an, Shaanxi, P.R. China; 2 Department of Respiratory and Critical Care Medicine, Bainiaohu Branch of People's Hospital of Xinjiang Uygur Autonomous Region, Urumqi, Xinjiang, P.R. China; 3 Department of Health Management, The Second Affiliated Hospital of Xi’an Jiaotong University, Xi’an, Shaanxi, P.R. China

**Keywords:** Yes-associated protein, glycolysis, proliferation, pulmonary hypertension, signal transduction

## Abstract

Glycolytic shift is implicated in the pathogenesis of pulmonary arterial hypertension (PAH). 6-Phosphofructo-2-kinase/fructose-2,6-biphosphatase 3 (PFKFB3) has been identified as a key enzyme regulating glycolysis. However, the molecular mechanisms underlying PFKFB3 regulation and glycolysis reprogramming in PAH remain unclear. Here, primary cultured pulmonary arterial smooth muscle cells (PASMCs) and monocrotaline-induced PAH rats were used to investigate these unknown mechanisms. We found that PFKFB3 expression and PASMC glycolysis were significantly increased in high mobility group box 1 (HMGB1)–treated cells, accompanied by the dephosphorylation and nuclear translocation of Yes-associated protein (YAP) *via* Rho-associated protein kinase signaling. Activation of YAP then acted as a transcriptional coactivator in conjunction with transcriptional enhancer activator domain 1, bolstering the transcription of the crucial glycolytic enzyme PFKFB3, consequently amplifying PASMC glycolysis. Rho-associated protein kinase inhibition, YAP or PFKFB3 knockdown, or glycolysis blockage diminished HMGB1-induced PASMC proliferation. In rats with monocrotaline-induced PAH, interventions such as inhibiting HMGB1 with glycyrrhizin, suppressing YAP activation with verteporfin, and targeting PFKFB3 with 3-PO effectively halted PAH progression. Our findings suggest that targeting the HMGB1–YAP–PFKFB3–glycolytic pathway is a promising strategy for preventing and treating PAH.

Pulmonary arterial hypertension (PAH) is a life-threatening pulmonary vascular disease characterized by sustained elevation of pulmonary vascular resistance and pulmonary arterial pressure, ultimately leading to right heart failure and death (1). Persistent vasoconstriction, excessive pulmonary vascular remodeling, and thrombosis *in situ* are identified as major pathogenesis in PAH (2). Pulmonary vascular remodeling is a fundamental structural alteration in all types of PAH, and pulmonary arterial smooth muscle cell (PASMC) proliferation is demonstrated to play a prominent role in this process (3). Therefore, elucidating the mechanism of PASMC proliferation in pulmonary vascular remodeling and finding appropriate targets are critical for PAH management.

Aerobic glycolysis (also known as the Warburg effect) is a metabolic hallmark present in most cancer cells and is characterized by an excessive conversion of glucose to lactate, even in the presence of ample oxygen (4). This glycolytic shift potently promotes tumor cell proliferation and survival (5). Recent work has revealed that the glycolytic mechanism is also implicated in PAH pathogenesis (6, 7). Increased glucose uptake and lactate production have been detected in the lungs of rodents with experimental PAH and patients with idiopathic PAH (8, 9), suggesting enhanced glycolytic activity in PAH. Further studies have found that PASMCs rely heavily on glycolysis for increased growth (10), whereas suppressing PASMC glycolysis by pharmacological inhibition impairs PASMC proliferation and prevents PAH occurrence (11). Among numerous glycolytic regulators, 6-phosphofructo-2-kinase/fructose-2,6-bisphosphatase 3 (PFKFB3) is a critical engine that controls the rate of glycolysis (12). Recent studies have indicated that PFKFB3 expression is upregulated in PASMCs isolated from patients with PAH and in the lungs of rodent PAH models (13, 14). Smooth muscle–specific *PFKFB3*-knockout mice exhibit attenuated hypoxia-induced PAH, and PFKFB3 inhibitors alleviate pulmonary arterial remodeling by inhibiting PASMC proliferation (15). These findings suggest that PFKFB3 is a potential new therapeutic target for treating PAH; however, the detailed molecular mechanisms underlying PFKFB3 upregulation and glycolytic reprogramming in PAH remain to be elucidated.

Yes-associated protein (YAP) is a key downstream effector of the Hippo pathway that plays an important role in tissue regeneration and tumorigenesis by regulating cell proliferation, apoptosis, and differentiation (16). In mammalian systems, the Hippo signaling pathway inactivates and sequesters YAP to the cytoplasm *via* phosphorylation at serine 127. Conversely, when Hippo pathway kinases are inactive, YAP dephosphorylates and translocates from the cytoplasm to the nucleus, where it acts as a transcriptional coactivator to promote the target gene transcription (17). Studies have demonstrated that YAP is highly expressed in the lung tissues of PAH patients and animal models (18, 19). YAP blockade or deletion dramatically inhibits PASMC proliferation and PAH progression (20, 21). Interestingly, YAP was recently identified as a key metabolic hub in regulating glycolysis (22, 23). Active YAP promotes cell glycolysis featured with enhanced glucose uptake and high lactate levels (24). Further studies have indicated that YAP activation modulates glycolytic enzymes, including PFKFB3, to coordinate glycolysis (25, 26). However, the potential connection between YAP and PFKFB3-mediated glycolysis remains unclear, particularly in PAH. Moreover, the upstream signaling molecules that regulate YAP activity in PAH are also largely unknown.

High mobility group box 1 (HMGB1), a critical damage-associated molecular pattern, is released by damaged cells under certain stress conditions. Once secreted, the extracellular HMGB1 stimulates cell proliferation, migration, and differentiation (27). Previous studies have indicated that HMGB1 and its downstream signaling play crucial roles in the pathophysiology of PAH (28, 29). Circulating HMGB1 levels are increased in patients with PAH and animal models of PAH (30) and are positively correlated with disease severity (31). Furthermore, it has been confirmed that HMGB1 promotes proliferation, hypertrophy, and migration of PASMCs (32, 33); inhibition of HMGB1 suppresses PASMC proliferation and mitigates pulmonary vascular remodeling in PAH rat models (34, 35). Other studies have shown that HMGB1 triggers dephosphorylation and nuclear translocation of YAP, resulting in elevated cell proliferation in various non-PASMC cell types (36, 37, 38). Taken together, these observations support our hypothesis that HMGB1 plays a pivotal role in regulating YAP activation. This activation, in turn, boosts the transcription of the critical glycolytic enzyme PFKFB3, thereby fostering glycolysis and driving PASMC proliferation, ultimately contributing to PAH progression.

## Results

### HMGB1 induces YAP activation, PFKFB3 upregulation, glycolysis, and proliferation in PASMCs

**Figure 1:**
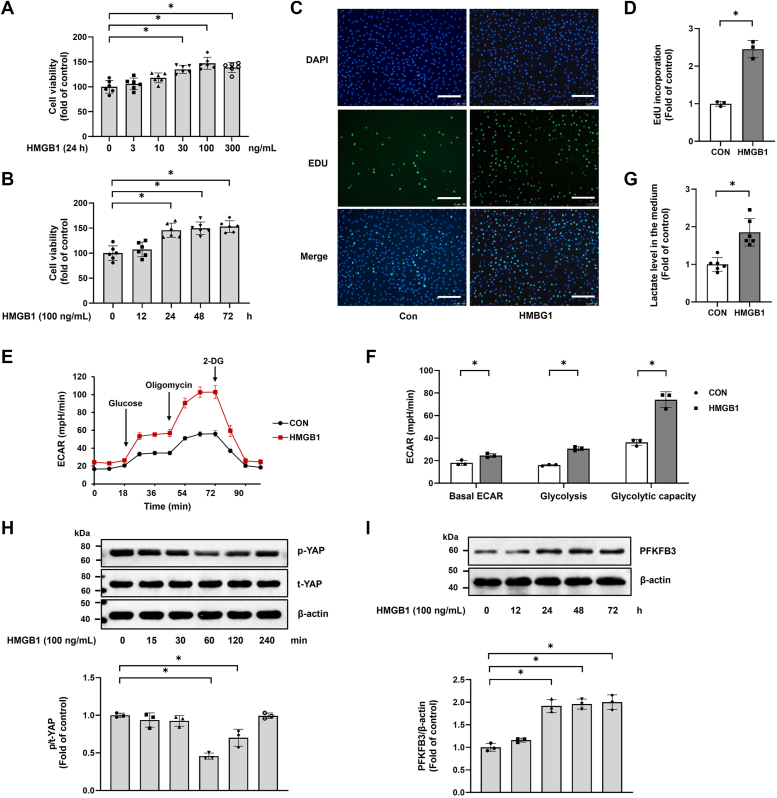
**HMGB1 induces YAP activation, PFKFB3 upregulation, glycolysis, and proliferation in PASMCs.***A*, PASMCs were incubated with HMGB1 for 24 h at concentrations ranging from 0 to 300 ng/ml. Cell viability was evaluated using the CCK-8 assay (n = 6 each group). *B*, PASMCs were stimulated with 100 ng/ml HMGB1 for different times (0, 12, 24, 48, and 72 h). Cell viability was evaluated using the CCK-8 assay (n = 6 each group). *C*, PASMCs were exposed to 100 ng/ml HMGB1 for 24 h. Cell proliferation was measured by EdU incorporation assay (the scale bar represents 100 μm) (n = 3 each group). *D*, quantitative analysis of EdU-positive cells. *E*, cell glycolysis was assessed by ECAR (n = 3). *F*, quantification of glycolytic function parameters of (*D*). *G*, lactate concentrations in culture medium were measured by ELISA (n = 6 each group). *H*, p-YAP and t-YAP levels were examined by Western blotting in PASMCs incubated with 100 ng/ml HMGB1 for the indicated time (0–120 min) (n = 3). *I*, PFKFB3 expression was examined by Western blotting in PASMCs with 100 ng/ml HMGB1 for the indicated time (0–72 h) (n = 3). ∗*p* < 0.05. Student’s *t* test was applied in *D*, *F*, and *G*. One-way ANOVA, multiple comparison, and Tukey’s post hoc test were applied for *A*, *B*, *H*, and *I*. CCK-8, Cell Counting Kit-8; ECAR, extracellular acidification rate; EdU, 5-ethynyl-2′-deoxyuridine; HMGB1, high mobility group box 1; PASMC, pulmonary arterial smooth muscle cell; PFKFB3, 6-phosphofructo-2-kinase/fructose-2,6-biphosphatase 3; YAP, Yes-associated protein.

First, we investigated the effect of HMGB1 on PASMC proliferation. The cells were exposed to different concentrations of HMGB1, ranging from 0 to 300 ng/ml for different times (0, 12, 24, 48, and 72 h). As shown in Figure 1*A*, HMGB1 dose-dependently stimulated PASMC proliferation. HMGB1 at a dose of 100 ng/ml caused the most notable increase in cell viability and was selected for subsequent cell experiments. Figure 1*B* depicts that HMGB1 promoted PASMC proliferation in a time-dependent manner. The results of the 5-ethynyl-2′-deoxyuridine (EdU) assay also demonstrated that the number of positive cells in the HMGB1 group (100 ng/ml) was significantly increased at 24 h compared with the control (Fig. 1, *C* and *D*). Furthermore, we evaluated the effect of HMGB1 on cell glycolysis by extracellular acidification rate (ECAR). The results indicated that intracellular metabolites of glycolysis (pyruvate, lactate, and 3-phosphoglycerate) were markedly increased in HMGB1-treated PASMCs (Fig. 1, *E* and *F*). Furthermore, lactate, the end product of aerobic glycolysis, also increased after HMGB1 treatment (Fig. 1*G*). Taken together, these results indicate that HMGB1 promotes PASMC proliferation and glycolysis.

To investigate the mechanisms underlying HMGB1-induced PASMC proliferation and glycolysis, we next explored the specific changes of YAP and PFKFB3 upon HMGB1 stimulation in PASMCs. As shown in Figure 1*H*, 100 ng/ml HMGB1 decreased p-YAP levels in a time-dependent manner, with a maximal effect observed at 1 h. However, total YAP expression remained unchanged. The protein level of PFKFB3 was also time-dependently upregulated upon HMGB1 stimulation (Fig. 1*I*). These results suggest that HMGB1 effectively induces YAP activation and PFKFB3 upregulation.

### Rho-associated protein kinase mediates HMGB1-induced YAP activation, PFKFB3 upregulation, and PASMC glycolysis

**Figure 2:**
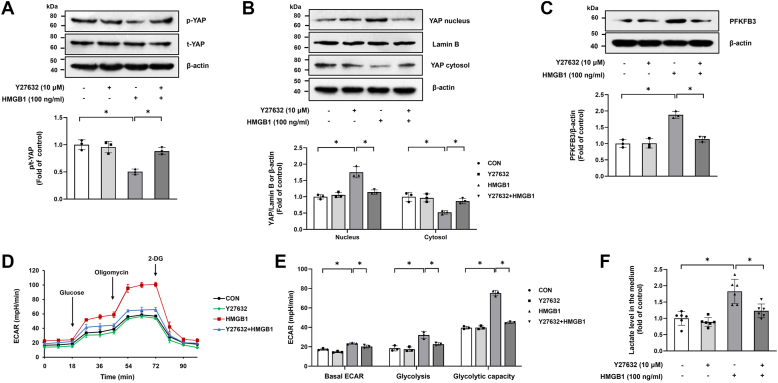
**ROCK mediates HMGB1-induced YAP activation, PFKFB3 upregulation, and PASMC glycolysis.***A*, PASMCs were pretreated with ROCK inhibitor Y27632 (10 μM) for 1 h and followed by stimulation with or without 100 ng/ml HMGB1 for another 1 h; p-YAP and t-YAP were determined by Western blotting (n = 3). *B*, PASMCs were pretreated with Y27632 (10 μM) for 1 h and followed by stimulation with or without 100 ng/ml HMGB1 for 24 h; protein levels of YAP in nuclear and cytoplasmic fractions were determined by Western blotting (n = 3). *C*, PFKFB3 expression was examined by Western blotting (n = 3). *D*, cell glycolysis was assessed by ECAR (n = 3). *E*, quantification of glycolytic function parameters of (*D*). *F*, lactate concentrations in culture medium were measured by ELISA (n = 6 each group). ∗*p* < 0.05. One-way ANOVA, multiple comparison, and Tukey’s post hoc test were applied for *A*, *B*, *C*, *E*, and *F*. ECAR, extracellular acidification rate; HMGB1, high mobility group box 1; PASMC, pulmonary arterial smooth muscle cell; PFKFB3, 6-phosphofructo-2-kinase/fructose-2,6-biphosphatase 3; ROCK, Rho-associated protein kinase; YAP, Yes-associated protein.

Previous studies, including ours, have revealed that YAP protein level and nuclear translocation are regulated by Rho-associated protein kinase (ROCK) signaling (39, 40). To investigate whether ROCK signaling mediates the effect of HMGB1 on YAP activation in PASMCs, cells were prior incubated with ROCK inhibitor Y27632 (10 μM, 1 h) and then stimulated with HMGB1. As shown in Figure 2*A*, HMGB1 effectively induced YAP dephosphorylation, whereas pretreatment of cells with Y27632 blocked HMGB1-induced dephosphorylation of YAP. Meanwhile, pretreatment of cells with ROCK inhibitor Y27632 notably decreased YAP protein level in the cell nucleus and increased YAP protein level in the cytosol compared with HMGB1-treated cells (Fig. 2*B*). These results suggest that HMGB1 acts *via* the ROCK pathway to induce YAP activation in PASMCs.

To further examine whether ROCK mediates HMGB1-induced PFKFB3 upregulation and PASMC glycolysis, cells were pretreated with Y27632 (10 μM, 1 h) before stimulation with HMGB1. As shown in Figure 2*C*, HMGB1 significantly upregulated PFKFB3 expression, whereas preincubation with Y27632 suppressed HMGB1-induced upregulation of PFKFB3. Figure 2, *D*–*F* indicates that inhibition of ROCK by Y27632 also notably mitigated HMGB1-induced PASMC glycolysis as indicated by lower levels of ECAR and less production of lactate. Together, these results reveal that HMGB1 upregulates PFKFB3 and promotes glycolysis through ROCK in PASMCs.

Extracellular HMGB1 has been shown to interact with multiple cell surface receptors, most notably, receptor for advanced glycation end products (RAGE) and Toll-like receptor 4 (TLR4), to promote inflammation and tumor growth (41). Our previous work has demonstrated that TLR4 majorly mediates HMGB1-induced PASMC proliferation and migration (33). We next investigated whether these receptors mediate HMGB1-induced YAP activation, PFKFB3 upregulation, and PASMC glycolysis. Pretreatment of cells with TLR4 inhibitor TAK-242 blocked HMGB1-induced YAP dephosphorylation and PFKFB3 upregulation, whereas pretreatment with RAGE inhibitor FPS-ZM1 did not significantly alter these changes caused by HMGB1 (Fig. S1, *A* and *B*). Moreover, inhibition of TLR4 attenuated HMGB1-induced PASMC glycolysis (Fig. S1, *C*–*E*). Collectively, these results suggest that HMGB1 promoted YAP activation, PFKFB3 upregulation, and PASMC glycolysis, primarily *via* TLR4 receptor.

### YAP mediates the effect of HMGB1 on PFKFB3 upregulation and PASMC glycolysis

**Figure 3:**
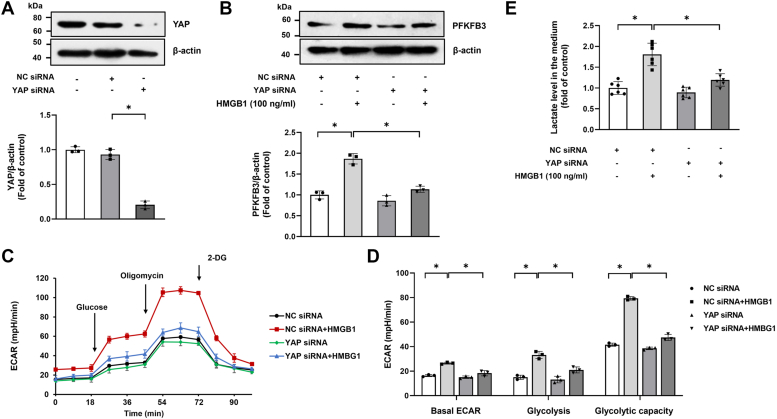
**YAP mediates the effect of HMGB1 on PFKFB3 upregulation and PASMC glycolysis.***A*, PASMCs were transfected with YAP siRNA or NC siRNA for 48 h, and the expression of YAP was examined by Western blotting (n = 3). *B*, PASMCs were pretransfected with YAP siRNA or NC siRNA for 24 h and followed by stimulation with or without HMGB1 for another 24 h; PFKFB3 expression was examined by Western blotting (n = 3). *C*, cell glycolysis was assessed by ECAR (n = 3). *D*, quantification of glycolytic function parameters of (*C*). *E*, lactate concentrations in culture medium were measured by ELISA (n = 6 each group). ∗*p* < 0.05. One-way ANOVA, multiple comparison, and Tukey’s post hoc test were applied for *A*, *B*, *D*, and *E*. ECAR, extracellular acidification rate; HMGB1, high mobility group box 1; NC, negative control; PASMC, pulmonary arterial smooth muscle cell; PFKFB3, 6-phosphofructo-2-kinase/fructose-2,6-biphosphatase 3; YAP, Yes-associated protein.

It has been reported that YAP plays a critical role in the regulation of glycolysis in a variety of tumor cells as well as nonmalignant cells (22, 23). To investigate whether YAP is involved in HMGB1-induced PFBFK3 upregulation and PASMC glycolysis, YAP was silenced using sequence-specific siRNA. Figure 3*A* shows that there was a prominent reduction in YAP expression after knockdown, and loss of YAP blocked HMGB1-induced upregulation of PFKFB3 (Fig. 3*B*). Figure 3, *C*–*E* demonstrates that the ECAR and lactate levels were declined in cells prior transfected with YAP siRNA. These results suggest that YAP mediates HMGB1-induced upregulation of PFBFK3 and PASMC glycolysis.

### YAP combines with transcriptional enhancer activator domain **1** and enhances PFKFB3 transcription

**Figure 4:**
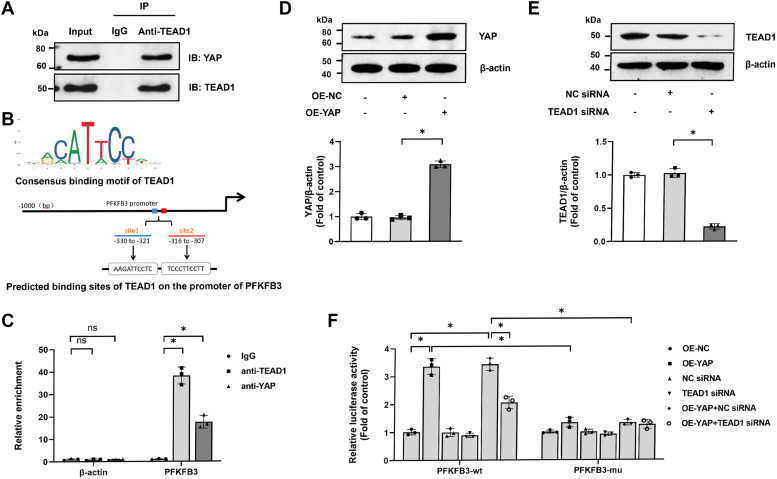
**YAP combines with TEAD1 and enhances PFKFB3 transcription.***A*, co-IP assay was performed to examine the interaction of YAP with TEAD1 in PASMCs (n = 3). *B*, consensus binding motif of TEAD1 and the binding sites of TEAD1 on the promoter of PFKFB3 were predicted using JASPAR database. *C*, chromatin immunoprecipitation (ChIP) assay was used to investigate the binding of TEAD1 or YAP to PFKFB3 or β-actin promoter in PASMCs. IgGs were used as a negative control (n = 3). The efficiencies of YAP overexpression (*D*) and TEAD1 knockdown (*E*) were evaluated by Western blotting after transfection for 48 h (n = 3). *F*, PASMCs were cotransfected with recombinant vector (wildtype or mutant-type PFKFB3 reporter vector) and overexpression plasmid (OE-YAP or OE-NC) with or without TEAD1 siRNA; luciferase assay was performed 48 h after transfection (n = 3). ∗*p* < 0.05. ns, not significant. One-way ANOVA, multiple comparison, and Tukey’s post hoc test were applied for *C*, *D*, *E*, and *F*. co-IP, coimmunoprecipitation; PASMC, pulmonary arterial smooth muscle cell; PFKFB3, 6-phosphofructo-2-kinase/fructose-2,6-biphosphatase 3; TEAD1, transcriptional enhancer activator domain 1; YAP, Yes-associated protein.

Lacking a DNA-binding domain, YAP depends on interaction with other transcription factors, particularly transcriptional enhancer activator domain 1 (TEAD1), for recruitment to chromatin and regulation of the transcription of its target genes (42). We first tested the existence of the YAP–TEAD1 complex in PASMCs. As shown in Figure 4*A*, a direct interaction between YAP and TEAD1 was confirmed by coimmunoprecipitation assay. To determine whether the YAP–TEAD1 complex directly regulates the transcription of PFKFB3, we surveyed JASPAR (http://jaspar.genereg.net/) and found two proximate putative TEAD1-binding sites within the promoter region of PFKFB3 (Fig. 4*B*). When precipitated with anti-TEAD1 or anti-YAP antibody, we found that the PFKFB3 promoter was co-occupied by TEAD1 and YAP (Fig. 4*C*). In addition, a luciferase reporter assay showed that the wildtype PFKFB3 promoter, but not the mutant constructs, was activated by YAP overexpression (OE), whereas transfection with TEAD siRNA significantly attenuated YAP OE–induced PFKFB3 promoter luciferase activity (Fig. 4*F*). Taken together, these data indicate that YAP–TEAD1 complexes bind to PFKFB3 promoter and directly activate its transcription in PASMCs. The efficiencies of YAP OE and TEAD1 knockdown are shown in Figure 4, *D* and *E*, respectively.

### PFKFB3 is required for HMGB1-induced glycolysis in PASMCs

**Figure 5:**
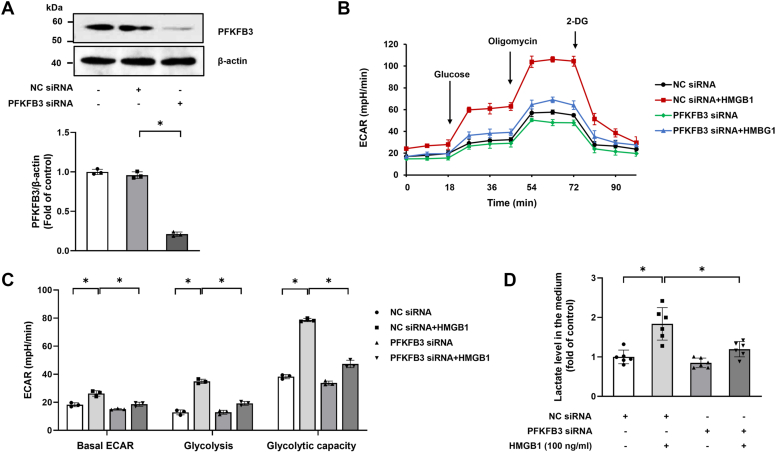
**PFKFB3 is required for HMGB1-induced glycolysis in PASMCs.***A*, PASMCs were transfected with PFKFB3 siRNA or NC siRNA for 48 h, and the expression of PFKFB3 was examined by Western blotting (n = 3). *B*, PASMCs were pretransfected with PFKFB3 siRNA or NC siRNA for 24 h and followed by stimulation with or without HMGB1 for another 24 h; cell glycolysis was measured by ECAR (n = 3). *C*, quantification of glycolytic function parameters of (*B*). *D*, lactate concentrations in culture medium were measured by ELISA (n = 6 each group). ∗*p* < 0.05. One-way ANOVA, multiple comparison, and Tukey’s post hoc test were applied for *A*, *C*, and *D*. ECAR, extracellular acidification rate; HMGB1, high mobility group box 1; NC, negative control; PASMC, pulmonary arterial smooth muscle cell; PFKFB3, 6-phosphofructo-2-kinase/fructose-2,6-biphosphatase 3.

PFKFB3, a master activator of glycolysis, is associated with numerous proliferative diseases (12). To examine whether PFKFB3 mediates HMGB1-induced glycolysis in PASMCs, siRNA-mediated PFKFB3 knockdown was performed. The transfection efficiency of PFKFB3-siRNA is shown in Figure 5*A*. Figure 5, *B*–*D* indicates that PFKFB3 siRNA transfection significantly suppressed PASMC glycolysis as indicated by the decreased levels of ECAR and lower lactate production, suggesting that PFKFB3 specifically mediates HMGB1-induced glycolysis in PASMCs.

### HMGB1 stimulates PASMC proliferation *via* the ROCK–YAP–PFKFB3–glycolysis pathway

**Figure 6:**
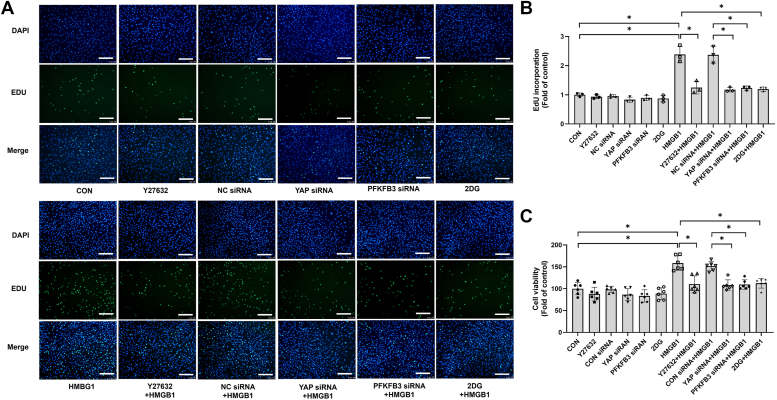
**HMGB1 stimulates PASMC proliferation *via* the ROCK–YAP–PFKFB3–glycolysis pathway.** PASMCs were pretreated with ROCK inhibitor Y27632 (10 μM, 1 h) or glycolysis inhibitor 2-DG (100 mM, 2 h) or pretransfected with YAP-siRNA, PFKFB3-siRNA, or NC siRNA for 24 h and followed by stimulation with or without HMGB1 for 24 h. Cell proliferation was detected by EdU incorporation assay (*A*) (the scale bar represents 100 μm) (n = 3 per group) and by CCK-8 assay (*C*) (n = 6 per group). *B*, quantitative analysis of EdU-positive cells. ∗*p* < 0.05. One-way ANOVA, multiple comparison, and Tukey’s *post hoc* test were applied for *B* and *C*. CCK-8, Cell Counting Kit-8; 2-DG, 2-deoxy-d-glucose; EdU, 5-ethynyl-2′-deoxyuridine; HMGB1, high mobility group box 1; PASMC, pulmonary arterial smooth muscle cell; PFKFB3, 6-phosphofructo-2-kinase/fructose-2,6-biphosphatase 3; ROCK, Rho-associated protein kinase; YAP, Yes-associated protein.

To further elucidate the role of ROCK, YAP, PFKFB3, and glycolysis in HMGB1-stimulated PASMC proliferation, PASMCs were incubated with ROCK inhibitor Y27632 or glycolysis inhibitor 2-deoxy-d-glucose (2-DG) or pretransfected with YAP-siRNA or PFKFB3-siRNA before stimulation with HMGB1. As shown in Figure 6, *A*–*C*, inhibition of ROCK, genetic deletion of YAP or PFKFB3, or blockage of glycolysis significantly reduced PASMC proliferation stimulated by HMGB1. Collectively, the results suggest that the ROCK–YAP–PFKFB3–glycolysis cascade mediates HMGB1-induced PASMC proliferation.

### Intervention of HMGB1, YAP, or PFKFB3 alleviates the development of PAH in monocrotaline-induced rats

**Figure 7:**
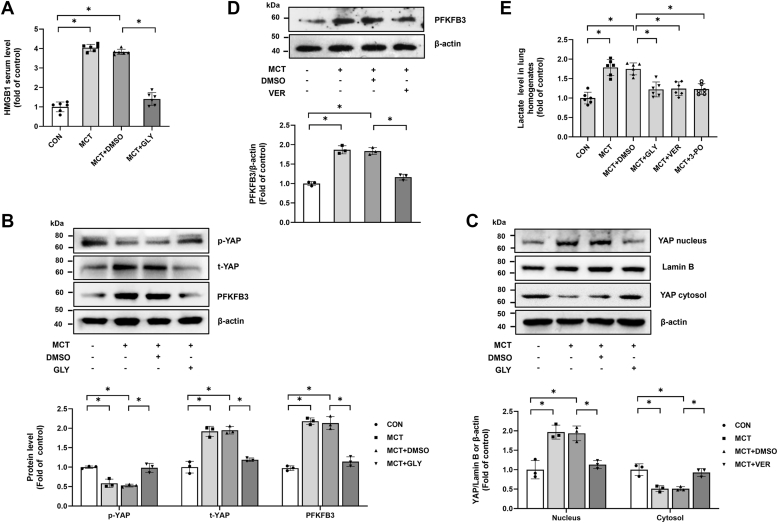
**Intervention of HMGB1, YAP, or PFKFB3 regulates relevant downstream target activity or expression in an MCT-induced rat model of PAH.***A*, HMGB1 serum levels in different groups were detected by ELISA. *B*, the protein levels of p-YAP, t-YAP, and PFKFB3 in lung tissues were measured using Western blotting. *C*, the protein levels of YAP in nuclear and cytoplasmic fractions in lung tissues were measured using Western blotting. *D*, PFKFB3 expression in lung tissues was assessed by Western blotting. *E*, lactate levels in lung homogenates in different groups were detected by ELISA. ∗*p* < 0.05. One-way ANOVA, multiple comparison, and Tukey’s post hoc test were applied for *A*, *B*, *C*, *D*, and *E*. HMGB1, high mobility group box 1; MCT, monocrotaline; PAH, pulmonary arterial hypertension; PFKFB3, 6-phosphofructo-2-kinase/fructose-2,6-biphosphatase 3; YAP, Yes-associated protein.

Based on the above *in vitro* experiments, we generated monocrotaline (MCT)-induced PAH rat model to further verify whether similar mechanisms are involved in the development of PAH. We first measured HMGB1 concentrations in serum and found that the serum HMGB1 level was increased in the MCT group compared with the control, whereas a significant decline was observed in the MCT group treated with the HMGB1 inhibitor glycyrrhizin (GLY) compared with the MCT group (Fig. 7*A*). We further showed that in the lungs of MCT-induced PAH rats, the phosphorylation level of YAP was decreased, the expression of YAP was raised, and the ratio of YAP protein levels in nucleus/cytosol was increased. However, GLY administration reversed the aforementioned alterations in MCT-induced PAH rats (Fig. 7, *B* and *C*). In addition, PFKFB3 expression was increased in rat PAH model, whereas administration of HMGB1 inhibitor GLY or YAP inhibitor verteporfin (VER) blunted this upregulation in MCT-induced rat PAH model (Fig. 7, *B* and *D*). Moreover, enhanced lactate concentrations in lung homogenates of MCT-induced PAH rats were also reduced by pharmacological inhibition of HMGB1, YAP, or PFKFB3 (Fig. 7*E*).

**Figure 8:**
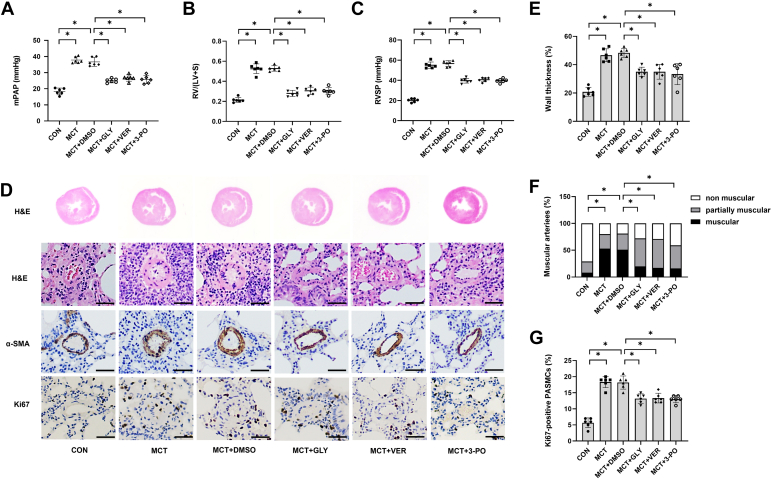
**Inhibition of HMGB1, YAP, or PFKFB3 alleviates the development of PAH in MCT-induced rats.***A*, changes of mPAP. *B*, changes of RVSP. *C*, changes of RV/(LV + S). *D*, RV hypertrophy shown by H&E staining. The medial wall thickness of pulmonary arterioles revealed by HE staining (the scale bar represents 100 μm); muscularization shown by α-smooth muscle actin (α-SMA) staining (the scale bar represents 100 μm); Ki67 staining showed PASMC proliferation (the scale bar represents 100 μm). *E*, quantitative analysis of the percentage of the medial wall thickness of pulmonary arteries. *F*, quantitative analysis of muscularization of pulmonary arterioles. *G*, quantitative analysis of Ki67-positive cells. ∗*p* < 0.05. One-way ANOVA, multiple comparison, and Tukey’s post hoc test were applied for *A*, *B*, *C*, *E*, *F*, and *G*. HMGB1, high mobility group box 1; LV, left ventricle; MCT, monocrotaline; mPAP, mean pulmonary arterial pressure; PAH, pulmonary arterial hypertension; PASMC, pulmonary arterial smooth muscle cell; PFKFB3, 6-phosphofructo-2-kinase/fructose-2,6-biphosphatase 3; RV, right ventricle; RVSP, right ventricular systolic pressure; S, interventricular septum; YAP, Yes-associated protein.

Finally, to explore the roles of HMGB1, YAP, and PFKFB3 in MCT-induced PAH in rats, PAH-related indicators were detected. As shown in Figure 8, *A* and *B*, inhibition of HMGB1, YAP, or PFKFB3 reduced the increase of mean pulmonary arterial pressure and right ventricular (RV) systolic pressure in MCT-induced PAH rats. In addition, the elevations of RV/(left ventricle [LV] + interventricular septum [S]), pulmonary arteriole medial wall thickness, proportion of muscularized arteries, and PASMC proliferation of pulmonary arterioles in MCT-induced PAH rats were also attenuated by treatment with HMGB1 inhibitor GLY, YAP inhibitor VER, and PFKFB3 inhibitor 3-PO, respectively (Fig. 8, *C*–*E*). Together, these results suggest that the HMGB1–YAP–PFKFB3-dependent glycolysis axis plays an important role in MCT-induced pulmonary vascular remodeling and PAH in rats.

## Discussion

This study has indicated that HMGB1-mediated YAP activation contributes to PFKFB3 upregulation and PASMC glycolysis, which promotes PASMC proliferation, pulmonary vascular remodeling, and ultimately the development of PAH. Thus, targeting this HMGB1–YAP–PFKFB3-dependent glycolytic pathway is a promising novel strategy for preventing and treating PAH.

HMGB1 belongs to a family of highly conserved chromosome proteins that contain HMG box domains (43). In addition to its nuclear function as a DNA chaperone, HMGB1 can be released by cells into the extracellular environment in response to various stimuli and functions as a key molecule in innate immunity, inflammation, and tissue remodeling (43, 44, 45). Abnormal HMGB1 expression and release have been found to be implicated in multiple diseases (46). Previous studies have demonstrated its involvement in PAH pathogenesis (28, 34, 35). Our present study showed that exogenous HMGB1 stimulated PASMC proliferation *in vitro*; serum HMGB1 levels were increased in the MCT-induced rat PAH model, and pharmacological inhibition of HMGB1 attenuated pulmonary vascular remodeling and ameliorated pulmonary hypertension, which are consistent with previous studies (28, 34, 35).

YAP, the nuclear effector of the Hippo pathway, is predominantly kept inactive in the cytoplasm through serine phosphorylation by the upstream mammalian Ste20-like kinase 1/2–large tumor suppressor 1/2 kinase cascade. Once dephosphorylated, YAP shuttles into the nucleus and binds to transcription factors to activate target gene expression (17). YAP has robust and diverse biological functions, such as cell contact inhibition, mechanotransduction, proliferation, differentiation, and apoptosis. YAP dysregulation has been associated with a wide range of human diseases (16). Recent work has shown that activation of YAP contributes to PASMC proliferation and participates in PAH development (18, 19, 20). However, the potential extracellular ligands and coupled intracellular signaling cascades that regulate the YAP activity in PAH remain elusive. HMGB1 has been identified as an important regulator of YAP activation in the extracellular fluid, which induces YAP nuclear translocation leading to cell migration, proliferation, and tumor growth (36, 37, 38). Other studies have demonstrated that HMGB1 activates ROCK by the GTP-bound form of Rho (47, 48), and Rho–ROCK signaling activates YAP by inhibiting the Hippo pathway kinases large tumor suppressor 1/2 in several types of non-PASMCs (40, 49). In this study, we observed that inhibition of YAP by genetic disruption and chemical inhibitor suppressed PASMC proliferation and ameliorated pulmonary vascular remodeling. We further demonstrated that HMGB1 induced dephosphorylation and nuclear localization of YAP, and that inhibition of ROCK reversed the effect of HMGB1 on YAP, suggesting that HMGB1 acts through ROCK to induce YAP activation.

Increased glucose metabolism and reprogramming toward aerobic glycolysis are hallmarks of proliferative diseases, including cancer and PAH (6). Recent work has highlighted the pivotal role of YAP in cellular energy metabolism, particularly in glycolysis (22, 23). Cells with constitutively active YAP have been observed to quickly acidify their culture medium, indicating increased glucose utilization and glycolysis (50). Further studies have shown that YAP controls the expression of glucose transporters and glycolytic enzymes and actively regulates intracellular glycolytic programs (25, 26). In this study, we showed that knockdown of YAP mitigated HMGB1-tigerred PASMC glycolysis, and inhibition of YAP by VER suppressed glycolysis to blunt pulmonary vascular remodeling, suggesting the crucial role of YAP in PASMC glycolysis in PAH. Importantly, previous studies have indicated that glycolysis is required to sustain YAP function (51). When cell glycolysis is increased, YAP is fully active; whereas glucose metabolism is blocked or glycolysis is reduced, YAP transcriptional activity is decreased (52, 53). These findings propose the existence of a positive feedback loop between YAP and glycolysis, which potentially contribute to the continuous augmentation of glycolysis and disease progression. However, many critical questions regarding the interplay between YAP and glycolysis still wait to be answered, and whether the similar mechanisms exist in PAH need further investigation.

PFKFB3, a master activator of glycolysis, catalyzes the synthesis of fructose-2,6-bisphosphate, leading to allosteric activation of the rate-limiting glycolytic enzyme phosphofructokinase-1 and consequently glycolytic flux (12). PFKFB3 is overexpressed in several human cancers and is associated with poor prognosis (54), whereas PFKFB3 knockdown or inhibition substantially inhibits cell survival, growth, and invasiveness (55, 56). Recently, upregulation of PFKFB3 has been reported in PASMCs from PAH patients and animal models, and it participates in PAH development by promoting PASMC proliferation and pulmonary vascular remodeling (14, 15). Consistent with this, our study showed that PFKFB3 expression was increased in HMGB1-triggerred PASMCs and in an MCT-induced PAH rat model, accompanied by elevated glycolysis. Genetic deletion or chemical inhibition of PFKFB3 impaired PASMC glycolysis and attenuated PASMC proliferation and pulmonary vascular remodeling. Given the important functions of YAP in regulating glycolysis and modulating glycolytic regulators, we therefore focus on the regulatory role of YAP in PFKFB3. Our data demonstrated that YAP acted as a transcriptional coactivator in conjunction with TEAD1, a key transcription factor partner for YAP-dependent gene expression, to augment the transcription of PFKFB3. The use of VER, a suppressor of YAP activity by blocking YAP–TEAD interaction (57), led to a decrease of PFKFB3 expression and impeded PAH development. Taken together, these results suggest that elevated glycolysis in PAH is associated with YAP-mediated PFKFB3 upregulation *via* TEAD1.

## Conclusions

In conclusion, we have first shown that HMGB1 acts through ROCK to activate YAP, which works together with TEAD1 to enhance the transcription of the important glycolytic enzyme PFKFB3 and promotes PASMC glycolysis, thereby contributing to PASMC proliferation. Our results uncover a novel signaling cascade involved in PAH pathogenesis and further demonstrated its therapeutic potential, providing new insights for the prevention and treatment of PAH.

## Experimental procedures

### Cell culture and reagents

Primary PASMCs were isolated from pulmonary arteries of male Sprague–Dawley rats (100–120 g) as previously described (58, 59). Cells were cultured in high-glucose Dulbecco’s modified Eagle's medium (Gibco) containing 10% fetal bovine serum (Gemini Bio) and 100 U/ml penicillin–streptomycin. Cells were incubated in a humidified incubator with 5% CO_2_ at 37 °C and passaged using 0.25% trypsin (Invitrogen). For maintaining the PASMC phenotype, early-passage cells (passage 3–6) were used for all experiments, and cell purity was determined by immunostaining for α-smooth muscle actin (1:200 dilution; BM0002; Boster). PASMCs were serum-starved (1% fetal bovine serum–Dulbecco’s modified Eagle’s medium) overnight prior to each experiment. HMGB1 (0–300 ng/ml) (1690-HMB050; R&D Systems) was used to stimulate PASMCs. Y27632 (10 μM; 129830-38-2; Cayman Chemical) was applied to inhibit ROCK, and 2-DG (100 mM; S4701; Selleckchem) was employed to block glycolysis. TAK-242 (1 μM; HY-19370; MedChem Express) and FPS-ZM1 (10 μM; HY-11109; MedChem Express) were used to inhibit HMGB1 receptors TLR4 and RAGE, respectively. The concentrations of the compounds were selected based on previous studies (32, 39, 60).

### Cell proliferation assay

Cell proliferation was assessed using Cell Counting Kit-8 and EdU incorporation assay. PASMCs were plated in 96-well plates at a density of 5 × 10^3^ cells per well. After different treatments according to the experimental designs, Cell Counting Kit-8 reagent (1:10 dilution; KGA317; KeyGen Biotech) was added into each well and incubated for 3 h, and the absorbance was recorded at 450 nm using a microplate reader (Bio-Rad). For the EdU incorporation assay, cells were labeled with EdU (C0071S; Beyotime) for 4 h at 37 °C. EdU-positive cells were visualized under an inverted fluorescence microscope and quantified using ImageJ software (National Institutes of Health).

### Cell transfection

PASMCs were seeded in 6-well plates and grown to 30% to 50% confluence. Then, cells were transfected with Lipofectamine 3000 reagent (Invitrogen) according to the manufacturer’s protocol. All siRNAs and plasmids used in this research were synthesized by GenePharma. The sequences of siRNA duplexes specific for YAP, PFKFB3, TEAD1, and negative control (NC) were:

YAP siRNA, sense 5′-CUGCCACCAAGCUAGAUAATT-3′, antisense 5′-UUAUCUAGCUUGGUGGCAGTT-3′; PFKFB3 siRNA, sense 5′-UCUUCACACCGUCCUGAAATT-3′, antisense 5′-UUUCAGGACGGUGUGAAGATT-3′; TEAD1 siRNA: sense 5′-GGAAACAAGUAGUAGAAAATT-3′, antisense 5′-UUUUCUACUACUUGUUUCCTT-3′; NC siRNA, sense 5′-UUCUCCGAACGUGUCACGUTT-3′, antisense 5′-ACGUGACACGUUC GGAGAAT-3′.

### Metabolic measurements

The ECAR was analyzed using an XF 96 Extracellular Flux Analyzer (Seahorse Bioscience) according to the manufacturer’s instructions. Briefly, PASMCs were seeded at a density of 3 × 10^4^ cells per well in XF96 microplates and incubated in a non-CO_2_ incubator for 2 h. After the probes were calibrated, glucose (10 mM), mitochondrial/ATP synthase inhibitor oligomycin (1 μM), and 2-DG (100 mM) were sequentially injected into each well at 20 min, 50 min, and 80 min. Data were assessed by Seahorse XF96 Wave software. The levels of lactate in the cell culture supernatants and in lung homogenates were measured with the Lactate Assay Kit (Jiancheng Bio) according to the manufacturer’s instructions.

### Western blotting analysis

Proteins were obtained from harvested cultured PASMCs and lung tissues using radioimmunoprecipitation assay lysis buffer (Beyotime) and separated by 10% to 15% SDS-PAGE. After being transferred to polyvinylidene difluoride membranes, membranes were probed with the following primary antibodies against: p-YAP(Ser127, 1:1000 dilution, 13008; Cell Signaling Technology), t-YAP (1:1000 dilution, 14074; Cell Signaling Technology), PFKFB3 (1:1000 dilution, Ab181861; Abcam), TEAD1 (1:1000 dilution, Ab133533; Abcam), Lamin B (1:1000 dilution, 12987-1-AP; Proteintech Group), and β-actin (1:1000 dilution, YM3028; Immunoway) at 4 °C overnight, and then reblotted with the secondary antibodies (anti-mouse, EK010, 1:5000 dilution; anti-rabbit, EK020, 1:5000 dilution; Zhuangzhi Bio) at room temperature for 1 h. Reactions were visualized with SuperSignal West Pico Chemiluminescent Substrate (Pierce Biotechnology) and then exposed to the autoradiographic film. Signaling was quantified from scanned films using Quality One software (Bio-Rad).

### Coimmunoprecipitation assay

Proteins were extracted using total protein extraction buffer (Beyotime), then incubated with Protein A/G agarose beads (ThermoFisher Scientific) and TEAD1 antibody overnight at 4 °C on a spinning wheel. The bead–antibody complexes were washed three times with extraction buffer and collected by centrifugation at 3000*g*. After boiling for 5 min, the immunoprecipitates were probed using Western blotting with the indicated primary antibodies.

### Chromatin immunoprecipitation assay

The chromatin immunoprecipitation assay was performed using the EpQuik Kit (Epigentek) according to the manufacturer’s instructions. Cells were fixed with 1% formaldehyde and then decrosslinked with 1.25 M glycine. After sonicating the chromatin into fragments (0.5–1 kb), the lysate was incubated with IgG (3420; Cell Signaling Technology) or specific antibodies against TEAD1 or YAP. The chromatin DNA was purified and subjected to quantitative RT–PCR detection on the Applied Biosystems StepOnePlus Real-Time PCR System (ThermoFisher Scientific) using TB Green Premix Ex Taq II (RR820A; TaKaRa). The data were analyzed using the 2^−ΔΔCt^ method. The primers (Sangon Biotech) for PFKFB3 and β-actin promoters are PFKFB3: Forward: 5′-GTGGCTATACAAGTCAAGCAG CG-3′, Reverse: 5′-TGAGCCGAGAGCCCT AGAGGT-3′; β-actin: Forward: 5′-TTCAATCCAGACCCCGTGTG-3′, Reverse: 5′-GCCCAGATGGACAAGAATAGCC-3′.

### Luciferase reporter assay

The fragment of wildtype PFKFB3-3′UTR, containing the predicted binding sites of TEAD1, and its mutant type were obtained and cloned downstream of the luciferase reporter in the pGL3-Basic vector by GenePharma. For the luciferase reporter assay, PASMCs were seeded into 24-well plates and then cotransfected with recombinant vector (wildtype or mutant-type reporter vector) and OE plasmid (OE-YAP or OE-NC) with or without TEAD1 siRNA using Lipofectamine 3000 (Invitrogen). After 48 h, cell extracts were prepared, and luciferase activities were measured by a dual-luciferase assay kit (Promega) according to the manufacturer's protocol. Renilla luciferase activity was used to adjust for variations in transfection and harvest efficiencies across experiments.

### Animal experiments

All animal experiments were conducted in strict accordance with the Guide for the Care and Use of Laboratory Animals of Xi’an Jiaotong University Animal Experiment Centre. The protocols used in this study were approved by the Laboratory Animal Care Committee of Xi’an Jiaotong University. Male Sprague–Dawley rats (190–210 g) were obtained from Xi’an Jiaotong University Experimental Animal Center. All rats were housed in a temperature-controlled room (20 ± 2 °C) with a 12 h light–dark cycle and were fed a standard diet. The rats were randomly divided into six groups (n = 8) and treated as follows: the control group received saline by a single intraperitoneal (ip) injection on day 1, and then with an equal volume of vehicle (0.9% NaCl) alone for 28 days; MCT group received a single ip injection of 60 mg/kg MCT (Must Bio-Technology) at day 1 to induce PAH; MCT + dimethyl sulfoxide group received vehicle dimethyl sulfoxide by daily ip injection; MCT + GLY group received GLY (100 mg/kg, 53956-04-0; Santa Cruz) by daily ip injection; MCT + VER group received VER (4.5 mg/kg, S1786; Selleckchem) by ip injection every 48 h; MCT + 3-PO group received 3-PO (25 mg/kg, S7639; Selleckchem) by ip injection once a day. The doses of all intervention reagents used in this study were based on previous experimental studies (15, 33, 61).

### Hemodynamic and RV hypertrophy measurements

After 4 weeks of intervention, rats were anesthetized by ip injection of 2% sodium pentobarbital for hemodynamic assessments as described previously (35). Mean pulmonary arterial pressure and RV systolic pressure were assessed through RV catheterization. To evaluate RV hypertrophy, the RV and LV plus S were dissected and measured to calculate the RV/LV + S ratio.

### Histological and immunohistochemistry staining

The heart and lung specimens were collected and fixed in 4% formalin, then embedded in paraffin, and sliced longitudinally at a thickness of 5 μm. Slides were stained with H&E following established protocols (32). Pulmonary arteriole vascular remodeling was assessed by measuring the media wall thickness of pulmonary arterioles (with diameters ranging between 50 and 200 μm, n = 10 per rat) by a light microscope (cellSens Imaging Software; Olympus) as documented (33). Immunohistochemistry staining for α-smooth muscle actin (1:200 dilution, BM0002; Boster) and Ki67 (1:200 dilution, YM3064; Immunoway) was conducted to detect pulmonary arteriole muscularization and PASMC proliferation as previously described (33).

### Statistical analysis

Statistical analyses were performed using SPSS, version 22.0 (IBM). All data are presented as mean ± standard deviation. Comparisons between two groups were analyzed by Student's *t* test, whereas comparisons of multiple groups were analyzed using one-way ANOVA with Tukey’s post hoc test. Statistical significance was set at *p* < 0.05 significance.

## Data availability

Data will be made available on request.

## Supporting information

This article contains supporting information.

## Conflict of interest

The authors declare that they have no conflicts of interest with the contents of this article.

## Figures and Tables

**Supplementary Figure:**
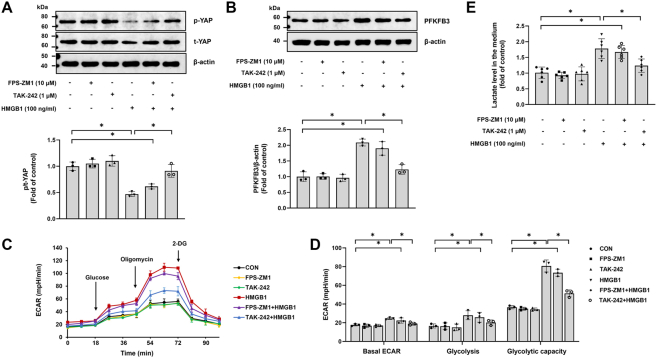
TLR4 majorly mediates HMGB1-induced YAP dephosphorylation, PFKFB3 upregulation, and PASMCs proliferation and glycolysis. A, PASMCs were pre-treated with FPS-ZM1 (10 μM) or TAK-242 (1 μM) for 30 min, and followed by stimulation with or without 100 ng/m L HMGB1 for 1 h, p-YAP and t-YAP were determined by western blotting (n = 3). PASMCs were pre-treated with FPS-ZM1 (10 μM) or TAK-242 (1 μM) for 30 min, and followed by stimulation with or without 100 ng/m L HMGB1 for 24 h, PFKFB3 level was examined by western blotting (B) (n = 3). C, Cells glycolysis was assessed by ECAR (n = 3). D, Quantification of glycolytic function parameters of (D). E, Lactate concentrations in culture medium were measured by ELISA (n = 6 each group). ∗*p* < 0.05. One-way ANOVA, Multiple comparison, Tukey’s post-hoc test were applied for A, B, D, E.
