# Supplementary material for: YAP-mediated glycolysis promotes pulmonary arterial smooth muscle cell proliferation in pulmonary arterial hypertension
Source: J Biol Chem. 2025 Oct 16;301(12):110836. doi: 10.1016/j.jbc.2025.110836 (PMC12664026; doi:10.1016/j.jbc.2025.110836)
Supplement: Supplementary Figure [file mmc1.docx]

**Figure legend**

**Supplementary Figure 1.** TLR4 majorly mediates HMGB1-induced YAP dephosphorylation, PFKFB3 upregulation, and PASMCs proliferation and glycolysis. A, PASMCs were pre-treated with FPS-ZM1 (10 μM) or TAK-242 (1 μM) for 30 min, and followed by stimulation with or without 100 ng/m L HMGB1 for 1 h, p‐YAP and t-YAP were determined by western blotting (n = 3). PASMCs were pre-treated with FPS-ZM1 (10 μM) or TAK-242 (1 μM) for 30 min, and followed by stimulation with or without 100 ng/m L HMGB1 for 24 h, PFKFB3 level was examined by western blotting (B) (n = 3). C, Cells glycolysis was assessed by ECAR (n = 3). D, Quantification of glycolytic function parameters of (D). E, Lactate concentrations in culture medium were measured by ELISA (n = 6 each group). **p* < 0.05. One-way ANOVA, Multiple comparison, Tukey’s post-hoc test were applied for A, B, D, E.
